# *S*. Enteritidis and *S*. Typhimurium Harboring SPI-1 and SPI-2 Are the Predominant Serotypes Associated With Human Salmonellosis in Saudi Arabia

**DOI:** 10.3389/fcimb.2019.00187

**Published:** 2019-05-31

**Authors:** Majed F. Alghoribi, Michel Doumith, Maha Alrodayyan, Maha Al Zayer, Wolfgang L. Köster, Abdulhai Muhanna, Sameera M. Aljohani, Hanan H. Balkhy, Taseen S. Desin

**Affiliations:** ^1^King Saud Bin Abdulaziz University for Health Sciences, Riyadh, Saudi Arabia; ^2^Infectious Diseases Research Department, King Abdullah International Medical Research Center, Riyadh, Saudi Arabia; ^3^Vaccine and Infectious Disease Organization–International Vaccine Center, University of Saskatchewan, Saskatoon, SK, Canada; ^4^John H. Stroger Junior Hospital of Cook County, Chicago, IL, United States; ^5^Ministry of National Guard-Health Affairs, Riyadh, Saudi Arabia

**Keywords:** Salmonella, SP-1, SPI-2, Type 3 secretion systems, serotyping

## Abstract

Non-typhoidal *Salmonella* (NTS) strains are Gram negative bacterial pathogens that are associated with foodborne illness worldwide. During the process of infection, *Salmonella* uses two molecular injectisomes known as Type 3 Secretion Systems (T3SS) to secrete virulence factors that are encoded by *Salmonella* Pathogenicity Island-1 (SPI-1) and SPI-2 into host cells. These secretion systems play a major role in virulence, as shown in various animal models, but little is known about their role in human infections. In Saudi Arabia, NTS strains frequently cause human infections but data regarding these pathogenic strains is fairly limited. The aim of this study was to characterize *Salmonella* human clinical isolates in Riyadh, Saudi Arabia, by determining their serotype, testing for the presence of SPI-1 and SPI-2 genes and to determine the antibiotic resistance profiles of these strains. Using the rapid Check and Trace *Salmonella*^™^ (CTS) system our results demonstrate that *S*. Enteritidis and *S*. Typhimurium were the predominant serovars, followed by *S*. Livingstone, *S*. Kentucky and *S*. Poona among a list of 36 serovars reported for the first time in the country. In addition, SPI-1 genes were detected in 99% of the isolates, while the *sifA* gene (SPI-2) was not detected in 13.5% of the isolates. These results suggest that both the SPI-1 and SPI-2 virulence determinants are important for human infection. Moreover, we report the presence of a Multi-Drug (MDR) carbapenem resistant *S*. Kentucky isolate harboring the *bla*_OXA−48_ gene not reported previously in Saudi Arabia.

## Introduction

*Salmonella enterica* is a Gram negative facultative intracellular bacterial pathogen that is capable of causing disease in a variety of hosts. *Salmonella enterica* consists of more than 2600 serovars of which *S*. *enterica* subspecies *enterica* is comprised of serovars like *S*. Typhi that are host-specific, while others like *S*. Typhimurium are generalists (Porwollik et al., 2004; Gal-Mor, 2019). Typhoid fever and invasive disease are typical symptoms that are associated with human infections caused by *S*. Typhi (Dougan and Baker, 2014). Non-typhoidal *Salmonella* (NTS) species mostly cause self-limiting gastrointestinal illness which may lead to hospitalization (requiring antibiotic treatment) and death. However, in sub-Saharan Africa, NTS species cause invasive disease (iNTS) that manifests as bacteremia in 8–45% of all community-acquired blood stream infections resulting in an overall case fatality rate of 20.6% (Haselbeck et al., 2017; Uche et al., 2017). The main source of NTS infections are the consumption of contaminated poultry products, though recently many outbreaks have been linked to fresh produce (Hanning et al., 2009). The global burden of NTS is estimated at 93.8 million cases of human infections resulting in 155,000 deaths annually (Ao et al., 2015). According to the CDC, NTS species are the second leading cause of foodborne illness in the U.S. which is estimated at 1.2 million annual cases of Salmonellosis. This results in 19,336 hospitalizations, 17,000 quality adjusted life years lost and USD $3.3 billion in total medical expenditures and lost productivity annually (Cummings et al., 2012). In addition, *S*. Enteritidis and *S*. Typhimurium are the most common serotypes associated with human infection in Africa, Asia, Europe, Latin America and the Caribbean, and in North America (Galanis et al., 2006). However, in Saudi Arabia very little is known about the prevalence of *Salmonella* serovars or their virulence properties associated with human infection. This information is vital for determining the source of these infections in order to develop intervention strategies aimed at reducing the levels of *Salmonella* species across the country (Gast, 2007). Hence, NTS human infections translate into a significant impact on both the healthcare system and the economy of a given country (Ghunaim and Desin, 2015).

During the process of infection, *Salmonella* uses two specialized nanomachines known as Type 3 Secretion Systems (T3SS) that are encoded by *Salmonella* Pathogenicity Island-1 (SPI-1) (Collazo and Galán, 1997) and SPI-2 (Hensel, 2000), respectively, to inject virulence factors directly into host cells. Traditionally, the SPI-1 T3SS has been associated with bacterial invasion of the host intestinal epithelial cell surface, while the SPI-2 T3SS has been linked to intracellular survival and maintenance of the *Salmonella* Containing Vacuole (SCV) (Galán, 2001). However, there has been recent evidence suggesting that there is a certain level of cross-talk between the two T3SS, indicating that the two systems are closely linked in their virulence functions (Brown et al., 2005; Coburn et al., 2005; Coombes et al., 2005). Many *in*-*vitro* and *in-vivo* studies have shown that the two *Salmonella* T3SS are important virulence factors as the deletion of these systems results in attenuation (Zhang et al., 2002; Hapfelmeier et al., 2004; Raffatellu et al., 2005; Desin et al., 2009; Wisner et al., 2010; Buckner et al., 2011). In human infections, the roles of SPI-1 and SPI-2 have not been defined as clearly, though a study in China has indicated that clinical isolates from a food-borne disease outbreak of *S*. Senftenberg lacking SPI-1 were still able to cause gastroenteritis (Hu et al., 2008). Several genes that encode for T3SS components which consist of structural or secreted effectors including *invA* (SPI-1 structure), *sipA* (SPI-1 effector), *sopE2* (SPI-1 effector), *sifA* (SPI-2 effector), and *ssaR* (SPI-2 structure) have been used as markers to test for the presence of these pathogenicity islands (Hu et al., 2008).

The emergence of antibiotic resistant bacteria worldwide is a significant public health concern resulting in 23,000 deaths per year in the U.S. at a cost of over $20 billion (Centers for Disease Control and Prevention, 2013). Moreover, drug-resistant NTS have been classified as a serious threat by the CDC with reports of increasing resistance to ceftriaxone and ciprofloxacin which are used as first-line antibiotic treatment. Alternative antimicrobials such as aminoglycosides, folic acid pathway inhibitors or the carbapenems are used for treatment of resistant NTS infections (Frye and Jackson, 2013). Therefore, from a patient safety perspective, it is critical to monitor antibiotic resistance patterns associated with human *Salmonella* infections. The objective of this study was to determine the prevalence of *Salmonella* serovars associated with human infections in Saudi Arabia using the rapid CTS system and to determine if the isolates contain SPI-1 and SPI-2 as well as characterizing the associated antimicrobial resistance.

## Materials and Methods

### *Salmonella* Clinical Isolates

NTS clinical isolates (*n* = 200) collected between May 2015 and Nov 2017 were obtained from the clinical microbiology laboratory at King Fahad Hospital (National Guard Health Affairs, King Abdulaziz Medical City, Riyadh). Isolates were from patients that presented with a variety of clinical symptoms ranging from gastrointestinal illness to systemic infection and were used in accordance with the ethics approval from the KAIMRC Institution Review Board (IRBC/040/16, IRBC/265/17, and IRBC/0768/18).

### PCR and Sequencing

Gene amplifications were performed with the MegaMIX Blue master mix (Gel Company, USA) using bacterial genomic DNA extracted from overnight cultures with the MagNa Pure LC 2.0 nucleic acid purification system (Roche), unless stated otherwise. Sanger sequencing was performed using an ABI 3730xL Genetic Analyzer (Applied Biosystems) at the KAIMRC core facility.

### Molecular Serotyping

*Salmonella* isolates were serotyped with the rapid CTS system (Checkpoints, Netherlands) (Wattiau et al., 2008b). The strains were streaked on Tryptic Soy Agar (TSA). Genomic DNA was extracted by resuspending a single colony from an agar plate into 100 μl Lysis buffer (CTS Kit) and incubated at 99°C for 15 min. The samples were serotyped using the PCR based approach described in the CTS manual as published previously (Ferrato et al., 2017). The samples were probed using specific DNA probes that correspond to an array pattern analyzed by the CTS software which assigns a *Salmonella* serovar to the unknown sample based on the available serovars in the CTS database. Serotypes of seven isolates that were not recognized by the CTS system were identified using Multi-Locus Sequence Typing (MLST) as an alternative serotyping method (Achtman et al., 2012). Briefly, the seven housekeeping genes, *aroC, dnaN, hemD, hisD, purE, sucA*, and *thrA* were amplified and sequenced. Samples were assigned alleles, sequence types (ST), and serotypes using the Achtman MLST database in Enterobase (http://enterobase.warwick.ac.uk) (Alikhan et al., 2018).

### Detection of SPI-1 and SPI-2 Genes

In order to detect the presence of the two major SPIs in the *Salmonella* isolates as described previously (Hu et al., 2008), primers to amplify SPI-1 (*invA, sipA, sopE2*) and SPI-2 (*sifA* and *ssaR)* genes (Table 1) were designed using the NCBI Primer-BLAST tool corresponding to the *S*. Typhimurium LT2 genome. The reference strain, *S*. Typhimurium strain SL 1344 (provided by VIDO-InterVac, University of Saskatchewan, Canada) was used as a positive control. Randomly selected amplicons representing all the 5 genes were sequenced for further confirmation.

**Table 1:** Primers used in this study to detect antimicrobial resistance determinants and SPI genes.

**Target**	**Primer sequence (5^**′**^ to 3^**′**^)**	**Amplicon size (bp)**	**Tm^**°**^C**	**References**
**β- LACTAMASES**				
*bla_TEM−1_*	F: CAT TTT CGT GTC GCC CTT AT R:TCC ATA GTT GCC TGA CTC CC	793	55	Lynne et al., 2008
*bla_OXA−1_*	F: ATG AAA AAC ACA ATA CAT ATC R: AAT TTA GTG TGT TTA GAA TGG	830	55	Wang et al., 2017
*bla_OXA−48_*	F: TTG GTG GCA TCG ATT ATC GG R: GAG CAC TTC TTT TGT GAT GGC	743	54.5	Poirel et al., 2004
*bla_CTX−M−1 group_*	F: GGT TAA AAA ATC ACT GCG TC R: TTA CAA ACC GTT GGT GAC GA	873	55	Wang et al., 2017
*bla_CTX−M−2 group_*	F: ACC GAG CCC ACG CTC AA R: CCG CTG CCG GTT TTA TC	221	55	Wang et al., 2017
*bla_CTX−M−9 group_*	F: AGA GTG CAA CGG ATG ATG R: AGA GTT ACA GCC CTT CGG	868	55	Wang et al., 2017
*bla_CMY−1_*	F: GTG GTG GAT GCC AGC ATC C R: GGT CGA GCC GGT CTT GTT GAA	915	55	Wang et al., 2017
*bla_PSE−1/CARB−2_*	F: CGC TTC CCG TTA ACA AGT AC R: CTG GTT CAT TTC AGA TAG CG	420	58	Weill et al., 2006
*bla_SHV_*	F: ATG CGT TAT ATT CGC CTG T R: TGC TTT GTT ATT CGG GCC AA	750	54.5	Hujer et al., 2006
*bla_VIM_*	F: GAT GGT GTT TGG TCG CAT A R: CGA ATG CGC AGC ACC AG	390	52	Dolejska et al., 2016
*bla_IMP_*	F: GGAATAGAGTGGCTTAATTCT C R: GGTTTAATAAAACAACCCC	188	52	Dolejska et al., 2016
*bla_NDM_*	F: GGT TTG GCG ATC TGG TTT TC R: CGG AAT GGC TCA TCA CGA TC	622	54.5	Poirel et al., 2011
*bla_CITM_*	F: TGG CCA GAA CTG ACA GGC AAA R: TTT CTC CTG AAC GTG GCT GGC	462	64	Perez-Perez and Hanson, 2002
*bla_MOXM_*	F: GCT GCT CAA GGA GCA CAG GAT R: CAC ATT GAC ATA GGT GTG GTG C	520	64	Perez-Perez and Hanson, 2002
*bla_DHAM_*	F: AAC TTT CAC AGG TGT GCT GGG T R: CCG TAC GCA TAC TGG CTT TGC	405	64	Perez-Perez and Hanson, 2002
*bla_ACCM_*	F: AAC AGC CTC AGC AGC CGG TTA R: TTC GCC GCA ATC ATC CCT AGC	346	64	Perez-Perez and Hanson, 2002
*bla_EBCM_*	F: TCG GTA AAG CCG ATG TTG CGG R: CTT CCA CTG CGG CTG CCA GTT	302	64	Perez-Perez and Hanson, 2002
*bla_FOXM_*	F: AAC ATG GGG TAT CAG GGA GAT G R: CAA AGC GCG TAA CCG GAT TGG	190	64	Perez-Perez and Hanson, 2002
**TOPOISOMERASES**				
*gyrA*	F: TGG GCA ATG ACT GGA ACA R: GGT TGT GCG GCG GGA TA	431	55	Wang et al., 2017
*parC*	F: ATG AGC GAT ATG GCA GAG CG R: TGA CCG AGT TCG CTT AAC AG	413	62	Wang et al., 2017
**SPI-1 GENES**				
*invA*	F: CCA CTT ACT TCC AGT GCG GA R: AGT GCT CGT TTA CGA CCT GA	2113	60	This study
*sipA*	F: TTC ATC GCA TCT TTC CCG GT R: CAC GAA TCT TGC GGC GAA TC	2010	60	This study
*sopE2*	F: CAT CAG GAG GCA TTC TGA AGAT R: CAC TAT CCA CCC AGC ACT ACA	712	60	This study
**SPI-2 GENES**				
*sifA*	F: CGT GAC GTC TGA GAA AGC GT R: TCC TTA CCA ACT CCC CAA GGA	1000	59.5	This study
*ssaR*	F: ATA GCG TTC CAG GTC GTG TC R: GTA CCA ATT GCG CCA GTG TC	668	60	This study

### Antibiotic Resistance Determination

Minimum Inhibitory Concentrations (MICs) to Ampicillin (AMP), Amoxicillin/Clavulanic acid (AMC), Piperacillin/Tazobactam (TZP), Cefalotin (CF), Cefoxitin (FOX), Ceftazidime (CAZ), Ceftriaxone (CRO), Cefepime (FEP), Imipenem (IPM), Meropenem (MEM), Amikacin (AMK), Gentamicin (GEN), Ciprofloxacin (CIP), Tigecycline (TGC), Nitrofurantoin (NIT), and Trimethoprim/Sulfamethoxazole (SXT) were determined using the Antimicrobial Susceptibility Test (AST) card AST-N291 for Gram negative bacteria for VITEK^®^ 2 Systems (BioMérieux) as described elsewhere (MacLowry and Marsh, 1968; Tilton et al., 1973; Bobenchik et al., 2017). The MICs were interpreted according to Clinical and Laboratory Standards Institute (CLSI) breakpoints (CLSI M100-ED28, 2018).

### Amplification of Antibiotic Resistance Genes

The detection of antibiotic resistance determinants was performed by PCR and sequencing using primers (Table 1) and amplification conditions as described by others (Weill et al., 2006; Lynne et al., 2008; Wang et al., 2017). Genes encoding CTX-M enzymes of groups 1, 2, and 9, plasmid-mediated AmpC of groups ACC, CIT, DHA, ENT/ENC, FOX and MOX, carbapenemase NDM, OXA-48-like, IMP, VIM as well as TEM, SHV, OXA-1, PSE-1/CARB-2 were sought by PCR in isolates showing resistance to one of the tested β-lactams. Quinolone resistance determinant regions (QRDRs) from *gyrA* and *parC* genes were sequenced and analyzed for point mutations that confer ciprofloxacin resistance (Hopkins et al., 2005).

## Results

### *S*. Enteritidis and *S*. Typhimurium Are the Predominant Serotypes

The majority (99%, 198/200) of the *Salmonella* species tested in this study belonged to *S*. *enterica* subspecies *enterica* and were comprised of 36 different serotypes (Table 2). The serotyping was mainly determined by the CTS system, while serotypes of 7 isolates that were untypeable using this approach were inferred from MLST. *S*. Enteritidis (40%, 80/200) was by far the most frequently isolated serovar, followed to a lesser extent by *S*. Typhimurium (13.5%, 27/200), *S*. Livingstone (5%, 10/200), *S*. Kentucky, *S*. Poona (4.5%, 9/200), *S*. Agona and *S*. Newport (2.5%, 5/200), and *S*. Give (2%, 4/200). The remaining serotypes had a prevalence ranging from one to three isolates each. Serotypes identified by MLST were *S*. Corvallis (ST2542, 2/200), *S*. Telhashomer (ST2399, 1/200) and *S*. Obogu (ST3335, 2/200), while one isolate could not be matched to any serotype in the database (ST3774) and one could not be identified.

**Table 2:** Prevalence of *Salmonella* serotypes associated with Human infections in Riyadh (2015–2017).

***Salmonella* serotype**	***n* (%)**
*S*. Enteritidis	80 (40)
*S*. Typhimurium	27 (13.5)
*S*. Livingstone	10 (5)
*S*. Kentucky	9 (4.5)
*S*. Poona	9 (4.5)
*S*. Agona	5 (2.5)
*S*. Newport	5 (2.5)
*S*. Give	4 (2)
*S*. Chailey	3 (1.5)
*S*. Corvallis	3 (1.5)
*S*. Galiema	3 (1.5)
*S*. Ohio	3 (1.5)
*S*. Orion	3 (1.5)
*S*. Schwarzengrund	3 (1.5)
*S*. Gaminara	2 (1)
*S*. Kottbus	2 (1)
*S*. Minnesota	2 (1)
*S*. Muenchen	2 (1)
*S*. Obogu	2 (1)
*S*. Saintpaul	2 (1)
*S*. Urbana	2 (1)
*S*. Altona	1 (0.5)
*S*. Anatum	1 (0.5)
*S*. Blockley	1 (0.5)
*S*. Caen	1 (0.5)
*S*. Eastbourne	1 (0.5)
*S*. Haifa	1 (0.5)
*S*. Havana	1 (0.5)
*S*. Infantis	1 (0.5)
*S*. Manhattan	1 (0.5)
*S*. Mbandaka	1 (0.5)
*S*. Molade	1 (0.5)
*S*. Montevideo	1 (0.5)
*S*. Pomona	1 (0.5)
*S*. Sandiego	1 (0.5)
*S*. Telhashomer	1(0.5)
Non-*enterica* subspecies	2 (1)
Unknown	2 (1)

### Systemic Infection Was Mainly Associated With *S*. Enteritidis and *S*. Typhimurium

The human clinical isolates (*n* = 170) were mainly isolated from stool samples and were comprised of 34 serotypes, with *S*. Enteritidis (38.8%, 66/170) and *S*. Typhimurium (12.4%, 21/170) being the most common (Table 3). The remaining strains were from the blood (*n* = 16), urine (*n* = 7), wound (*n* = 4), abdominal fluid (*n* = 2) or tissue (*n* = 1). Interestingly, 11.3% (9/80) of the *S*. Enteritidis strains were isolated from the blood, while only 7.4 % (2/27) of the *S*. Typhimurium strains were isolated from the same site. In addition to the aforementioned serotypes, non-fecal isolates were also isolated from the urine (*S*. Poona), wound (*S*. Agona and *S*. Minnesota), and blood (*S*. Poona and *S*. Urbana). However, *S*. Livingstone and *S*. Kentucky, despite being the third and fourth most prevalent serovars (Table 2), were exclusively isolated from stool samples. Taken together, this may suggest that *S*. Enteritidis may have a higher tendency of causing system infection relative to the other serotypes.

**Table 3:** Prevalence of *Salmonella* serotypes based on the source of the clinical sample.

**Serotype**	***n***	**Stool**	**Urine**	**Tissue**	**Abdominal fluid**	**Wound**	**Blood**
Agona	5	4	0	0	0	1	0
Chailey	3	2	0	0	0	0	1
Corvallis	3	3	0	0	0	0	0
Enteritidis	80	66	2	0	2	1	9
Galiema	3	1	0	0	0	0	2
Gaminara	2	2	0	0	0	0	0
Give	4	4	0	0	0	0	0
Kentucky	9	9	0	0	0	0	0
Kottbus	2	2	0	0	0	0	0
Livingstone	10	10	0	0	0	0	0
Minnesota	2	1	0	0	0	1	0
Muenchen	2	2	0	0	0	0	0
Newport	5	5	0	0	0	0	0
Obogu	2	2	0	0	0	0	0
Ohio	3	3	0	0	0	0	0
Orion	3	3	0	0	0	0	0
Poona	9	7	1	0	0	0	1
Saintpaul	2	2	0	0	0	0	0
Schwarzengrund	3	3	0	0	0	0	0
Typhimurium	27	21	2	1	0	1	2
Urbana	2	1	0	0	0	0	1
Others^a^	15	13	2	0	0	0	0
Non-enterica	2	2	0	0	0	0	0
Unknown (ST3374)	2	2	0	0	0	0	0
Total	200	170	7	1	2	4	16

a *Others includes serotypes for which there was only one strain positive for that particular serotype: S. Altona, S. Anatum, S. Blockley, S. Caen, S. Eastbourne, S. Haifa, S. Havana, S. Infantis, S. Manhattan, S. Mbandaka, S. Molade, S. Montevideo, S. Pomona, S. Sandiego, and S. Telhashomer*.

### SPI Genes Were Present in the Majority of Clinical Isolates

The SPI-1 (*invA, sipA, sopE2*) and SPI-2 (*sifA, ssaR*) genes were present in 84.5% (169/200) of the *Salmonella* strains (Table 4). The pathogenicity island genes were not detected in 15.5% (31/200) of the isolates in the form of either a SPI-1 gene (1%, 2/200 isolates), SPI-2 gene (13.5%, 27/200 isolates) or both SPI-1 and SPI-2 genes (1%, 2/200 isolates). The *sopE2* gene (SPI-1) was absent in a single isolate each belonging to *S*. Schwarzengrund and *S*. Kentucky. In contrast, the SPI-2 gene, *sifA*, was not detected in 13.5% (27/200) strains corresponding to various serotypes. The *ssaR* gene (SPI-2) was present in all *S*. *enterica* subspecies tested, except for the non-*enterica* subspecies, which were also missing other SPI-1 and SPI-2 genes. Additionally, it is noteworthy to highlight that 97% (28/29) of the *Salmonella* isolates lacking the *sifA* gene were exclusively isolated from fecal samples suggesting that SPI-2 may be important in human systemic infection.

**Table 4:** *S*. *enterica* serotypes that lack SPI virulence genes.

**Serotype**	**Total strains found**	**SPI genes absent**	**Frequency of strains with a missing SPI gene**	**Total strains with a missing SPI gene for a serotype**
Caen	1	*sifA*	1/1 (100%)	1
Corvallis	3	*sifA*	2/3 (67%)	2
Enteritidis	80	*sifA*	2/80 (2.5%)	2
Havana	1	*sifA*	1/1 (100%)	1
Kentucky	9	*sopE2*	1/9 (11%)	8
		*sifA*	7/9 (78%)	
Livingstone	10	*sifA*	5/10 (50%)	5
Mbandaka	1	*sifA*	1/1 (100%)	1
Molade	1	*sifA*	1/1 (100%)	1
Obogu	2	*sifA*	1/2 (50%)	1
Ohio	3	*sifA*	3/3 (100%)	3
Orion	3	*sifA*	1/3 (33%)	1
Schwarzengrund	3	*sopE2*	1/3 (33%)	1
Typhimurium	27	*sifA*	1/27 (3.7%)	1
Non-enterica subspecies	2	*sipA, sopE2, sifA*	1/2 (50%)	1
		*sipA, sopE2, sifA, ssaR*	1/2 (50%)	1
Unknown	2	*sifA*	1/2 (50%)	1
Total	148			31

### Antibiotic Resistance Profiles of the *Salmonella* Strains

Overall, the *Salmonella* strains demonstrated the highest level of antibiotic resistance against nitrofurantoin (17%, 34/200) (Table 5) with the majority being associated with *S*. Enteritidis (88.2%, 30/34) (Table 6). This was followed by resistance to ampicillin (13.5%, 27/200), cefalotin (10%, 20/200), tigecycline (10%, 20/200), ciprofloxacin (8.5%, 17/200), and trimethoprim/sulfamethoxazole (8%, 16/200) (Table 5). Resistance to other β-lactams including cephalosporins and carbapenems, as well as aminoglycosides, was below 5%. However, a significant number of isolates (61.5%, 123/200) were fully susceptible to all the tested antibiotics (Table 6). Interestingly, 13 strains identified as *S*. Agona (*n* = 3), *S*. Blockley (*n* = 1), *S*. Enteritidis (*n* = 1), *S*. Havana (*n* = 1), *S*. Kentucky (*n* = 1), *S*. Minnesota (*n* = 2), *S*. Newport (*n* = 1), and *S*. Typhimurium (*n* = 3) were Multi-Drug Resistant (MDR) strains as they demonstrated resistance to more than three antibiotic classes. In addition, one single isolate of *S*. Kentucky conferred resistance to carbapenem (imipenem and meropenem) and to ten other antibiotics (AMP, AMC, TZP, CF, CAZ, CRO, FEP, CIP, TGC, and SXT) making this strain a unique MDR isolate in the region.

**Table 5:** Prevalence of antibiotic resistance among human clinical *Salmonella* isolates.

**Antibiotics**	**Number of Strains**	**Percentage (%)**
NIT	34	17
AMP	27	13.5
CF	20	10
TGC	20	10
CIP	17	8.5
SXT	16	8
GEN	9	4.5
AMC	7	3.5
CAZ	4	2
CRO	4	2
TZP	4	2
AMK	2	1
FOX	2	1
FEP	1	0.5
IPM	1	0.5
MEM	1	0.5

**Table 6:** Antibiogram for *Salmonella* strains that are resistant to antibiotics.

**Serotype**	**Antibiotic resistance pattern**	**Number of antibiotics**	**Frequency of strains for a given pattern**
Agona	CIP-TGC-SXT AMP-GEN-CIP-TGC-SXT AMP-AMC-TZP-CF-AMK-GEN-CIP-TGC-SXT	3 5 9	1 1 1
Blockley	CIP-TGC-NIT	3	1
Caen	TGC	1	1
Chailey	NIT	1	1
Enteritidis	AMP NIT AMP-CF CIP-NIT CIP-TGC CF-CIP AMP-CIP-NIT	1 1 2 2 2 2 3	1 28 1 1 1 1 1
Give	AMP-CF-CAZ-CRO-GEN	5	1
Haifa	AMP-CF-GEN	3	1
Havana	AMP-AMC-TZP-CF-CIP-SXT	6	1
Infantis	CF	1	1
Kentucky	GEN-CIP AMP-AMC-CF-GEN AMP-AMC-TZP-CF-CIP AMP-AMC-TZP-CF-CAZ-CRO-FEP-IPM-MEM-CIP-TGC-SXT	2 4 5 12	2 1 1 1
Livingstone	NIT GEN-SXT	1 2	1 1
Minnesota	AMP-AMC-CF-FOX-CAZ-CRO-CIP-TGC	8	2
Newport	TGC AMP-GEN-SXT	1 3	3 1
SaintPaul	AMP	1	1
Typhimurium	AMP NIT SXT TGC AMP-CF AMP-SXT AMP-CIP-SXT AMP-CF-SXT AMP-CF-TGC AMP-CF-CIP-TGC AMP-CF-TGC-SXT	1 1 1 1 2 2 3 3 3 4 4	1 1 1 4 1 3 1 4 1 1 1
Total			77

### Characterization of Resistance to β-Lactam Antibiotics

Twenty-seven isolates (13.5%) (Table 7), which were mainly associated with *S*. Typhimurium (41%, 11/27), that had resistance to at least one of the β-lactam antibiotics were tested for the presence of β-lactam genes. The majority (66.6%, 18/27) of these strains were only resistant to ampicillin and carried the *bla*_TEM−1_ (*n* = 16) or *bla*_PSE−1_ (*n* = 2) genes. Of the remainder, three isolates had resistance to ceftazidime of which two were also resistant to piperacillin/tazobactam and amoxicillin/clavulanic acid and carried the *bla*_CMY−2_ gene, while one isolate remained susceptible to the two β-lactam/β-lactam inhibitor combinations and harbored the *bla*_TEM−1_ and *bla*_CTX−M−1−group_ genes. In addition, three other isolates had resistance to the β-lactam/β-lactam inhibitors but remained susceptible to cephalosporins with one strain carrying the *bla*_OXA−1_ gene and the two others only containing the *bla*_TEM−1_ genes. The MDR *S*. Kentucky isolate described above that conferred resistance to all the β-lactam antibiotics, including carbapenem, contained the *bla*_CTX−M−9−group_ and *bla*_OXA−48−like_ carbapenemase genes.

**Table 7:** Genotypes for *Salmonella* strains with resistance to β-lactam antibiotics and Ciprofloxacin.

**Serotype**	**Strain ID**	**AMP**	**AMC**	**TZP**	**CF**	**FOX**	**CAZ**	**CRO**	**FEP**	**IPM**	**MEM**	**CIP**	**β-lactamase genes**	**GyrA**	**ParC**
Agona	117	< 2	< 2	< 4	4	< 4	< 1	< 1	< 1	< 0.25	< 0.25	>4^*^		S83F/D87N	T57S/S80I
	145	>32^*^	16	16	4	< 4	< 1	< 1	< 1	< 0.25	< 0.25	>4^*^	*bla*_OXA−1_	S83F/D87N	T57S/S80I
	189	>32^*^	>32^*^	>128^*^	16^*^	8	< 1	< 1	2	< 0.25	< 0.25	>4^*^	*bla*_OXA−1_	S83F/D87N	T57S/S80I
Blockley	3	8	< 2	8	4	8	< 1	< 1	< 1	< 0.25	< 0.25	>4^*^		S83F/D87G	T57S/S80R
Enteritidis	4	< 2	< 2	< 4	4	< 4	< 1	< 1	< 1	< 0.25	< 0.25	2^*^		S83Y	
	50	>32^*^	4	< 4	16^*^	< 4	< 1	< 1	< 1	< 0.25	< 0.25	< 0.25	*bla*_TEM−1_		
	118	4	< 2	< 4	4	< 4	< 1	< 1	< 1	< 0.25	< 0.25	1^*^		S83Y	
	121	>32^*^	4	< 4	4	< 4	< 1	< 1	< 1	< 0.25	< 0.25	< 0.25	*bla*_TEM−1_		
	161	>32^*^	8	< 4	4	< 4	< 1	< 1	< 1	< 0.25	< 0.25	1^*^	*bla*_TEM−1_	WT	
	191	8	< 2	< 4	8	< 4	< 1	< 1	< 1	< 0.25	< 0.25	1^*^		S83Y	
Give	154	>32^*^	4	< 4	>64^*^	< 4	>64^*^	>64^*^	4	< 0.25	< 0.25	< 0.25	*bla*_TEM−1_, *bla*_CTX−M−1−group_		
Haifa	173	>32^*^	4	< 4	8^*^	< 4	< 1	< 1	< 1	< 0.25	< 0.25	< 0.25	*bla*_TEM−1_		
Havana	87	>32^*^	>32^*^	>128^*^	>64^*^	8	< 1	< 1	< 1	< 0.25	< 0.25	1^*^	*bla*_TEM−1_	S83F	
Kentucky	14	< 2	< 2	< 4	2	< 4	< 1	< 1	< 1	< 0.25	< 0.25	>4^*^		S83F/D87N	T57S/S80I
	22	>32^*^	>32^*^	32	>64^*^	< 4	< 1	< 1	< 1	< 0.25	< 0.25	< 0.25	*bla*_TEM−1_	S83F/D87G	T57S/S80I
	35	>32^*^	>32^*^	>128^*^	>64^*^	16	16^*^	>64^*^	32^*^	8^*^	8^*^	>4^*^	*bla*_CTX−M−9−group_, *bla*_OXA−48_	S83F/D87G	
	61	>32^*^	>32^*^	>128^*^	>64^*^	16	2	< 1	< 1	< 1	< 0.25	< 0.25	*bla*_TEM−1_		
	179	< 2	< 2	< 4	2	< 4	< 1	< 1	< 1	< 0.25	< 0.25	>4^*^		S83F/D87N	T57S/S80I
Minnesota	76	>32^*^	>32^*^	< 4	>64^*^	>64^*^	16^*^	8^*^	< 1	< 0.25	< 0.25	1^*^	*bla*_CMY−2_	WT	
	95	>32^*^	>32^*^	< 4	>64^*^	>64^*^	16^*^	8^*^	< 1	0.5	< 0.25	1^*^	*bla*_CMY−2_	WT	
Newport	181	>32^*^	16	< 4	4	< 4	< 1	< 1	< 1	< 0.25	< 0.25	< 0.25	*bla*_PSE−1_		
Saintpaul	124	>32^*^	4	< 4	4	< 4	< 1	< 1	< 1	< 0.25	< 0.25	< 0.25	*bla*_TEM−1_		
Typhimurium	20	>32^*^	16	8	< 2	< 4	< 1	< 1	< 1	< 0.25	< 0.25	< 0.25	*bla*_PSE−1_		
	27	>32^*^	4	< 4	8	< 4	< 1	< 1	< 1	< 0.25	< 0.25	< 0.25	*bla*_TEM−1_		
	34	>32^*^	8	< 4	4	< 4	< 1	< 1	< 1	< 0.25	< 0.25	< 0.25	*bla*_TEM−1_		
	38	>32^*^	8	< 4	8^*^	< 4	< 1	< 1	< 1	< 0.25	< 0.25	>4^*^	*bla*_TEM−1_	S83F/D87G	S80I
	55	>32^*^	8	< 4	8^*^	< 4	< 1	< 1	< 1	< 0.25	< 0.25	< 0.25	*bla*_TEM−1_		
	59	>32^*^	8	< 4	8^*^	< 4	< 1	< 1	< 1	< 0.25	< 0.25	< 0.25	*bla*_TEM−1_		
	92	>32^*^	4	< 4	4	< 4	< 1	< 1	< 1	< 0.25	< 0.25	< 0.25	*bla*_TEM−1_		
	137	>32^*^	8	< 4	8^*^	< 4	< 1	< 1	< 1	< 0.25	< 0.25	< 0.25	*bla*_TEM−1_		
	163	>32^*^	8	< 4	8^*^	< 4	< 1	< 1	< 1	< 0.25	< 0.25	< 0.25	*bla*_TEM−1_		
	167	>32^*^	< 4	4	8^*^	< 4	< 1	< 1	< 1	< 0.25	< 0.25	< 0.25	*bla*_TEM−1_		
	184	>32^*^	8	< 4	4	< 4	< 1	< 1	< 1	< 0.25	< 0.25	< 0.25	*bla*_TEM−1_		
	190	>32^*^	8	< 4	8^*^	< 4	< 1	< 1	< 1	< 0.25	< 0.25	< 0.25	*bla*_TEM−1_		

* *Resistant to a given antibiotic as per CLSI standards*.

### Characterization of Genes Encoding Resistance to Ciprofloxacin

Sixteen (8%) *Salmonella* strains had reduced susceptibility to ciprofloxacin with MICs ranging from 1 to ≥ 4 μg/ml (Table 7). Resistance was primarily seen in *S*. Agona (60%, 3/5 isolates), *S*. Kentucky (40%, 4/10) and *S*. Enteritidis (5%, 4/80). In order to identify mutations encoding for ciprofloxacin resistance, PCR was performed on the *gyrA* and *parC* genes. Eight isolates with an MIC ≥ 4 μg/ml had mutations in GyrA (S83F/Y and D87G/N) and ParC (T57S and S80I/R) amino acid sequences. In contrast, five of the isolates with MICs between 1 and 2 μg/ml had only a single mutation in the GyrA (S83Y/F) amino acid sequence, while three strains had no detectable mutations.

## Discussion

NTS species are a public health concern as they are prevalent worldwide causing a significant number of human infections (Gal-Mor, 2019). Rapid identification of *Salmonella* species associated with human infections is a critical component of a *Salmonella* control program since this allows for epidemiological investigations that can assist in determining the source of the infections (Ferrato et al., 2017). In addition, the presence of important virulence factors such as SPI-1 and SPI-2 enhance our understanding of the virulence potential of these strains (Dos Santos et al., 2019). Similarly, characterization of antimicrobial resistance associated with these strains can further assist in reducing the spread of antibiotic resistance mechanisms within *Salmonella* species (Parisi et al., 2018). The aim of the present study was to serotype *Salmonella* species associated with human infections in Saudi Arabia using a rapid method (CTS system) and to characterize the clinical isolates by testing for the presence of SPI-1 and SPI-2 virulence determinants as well as determining antimicrobial resistance patterns.

We chose to use the CTS system since it is a rapid technique that can be used for serotyping *Salmonella* species with a turnaround time of 8 hours. This system has been shown to be highly effective and accurate in determining *Salmonella* serotypes (Wattiau et al., 2008a; Ferrato et al., 2017). The CTS system was able to identify 96.5% of the *Salmonella* clinical isolates used in the current study. This establishes a fast and reliable method for the identification of *Salmonella* species associated with human infections in Saudi Arabia and in the region. The remaining isolates were identified using MLST which has been used successfully for the serotyping of *Salmonella* species (Achtman et al., 2012). The results from MLST imply that some of the serotypes not identified by the CTS were rare serotypes like *S*. Obogu and *S*. Telhashomer that are not present in the CTS database. In addition, ST3774 and the strain that was not identified by MLST could possibly be novel serotypes that are associated with human infection in Saudi Arabia. Our results indicate that the predominant serovars associated with human infections in Riyadh are *S*. Enteritidis and *S*. Typhimurium, followed by *S*. Livingstone, *S*. Kentucky and *S*. Poona, respectively. This is the first report in the region which demonstrates that besides *S*. Enteritidis and *S*. Typhimurium being the leading cause of human infections, serotypes *S*. Livingstone, *S*. Kentucky, and *S*. Poona are also significant. Moreover, the data also show that 36 unique *Salmonella* serotypes were circulating among the human population in Riyadh (Saudi Arabia). The fact that *S*. Enteritidis and *S*. Typhimurium are the most common serotypes is in agreement with surveillance data from the U.S. reported by FoodNET (Marder Mph et al., 2018) and the European Union (Gal-Mor, 2019). These results are also supported by a preliminary study performed in Saudi Arabia where the authors examined 33 isolates which included Typhoidal *Salmonella* species (El-Tayeb et al., 2017). Our results differ with respect to the third, fourth and fifth most common serovars since FoodNET has indicated that they are *S*. Newport, *S*. Javiana and the monophasic variant of *S*. Typhimurium (I 4, [5],12:i:-) (Marder Mph et al., 2018), in decreasing order, while in the European Union they are monophasic *S*. Typhimurium, *S*. Infantis and *S*. Derby (Gal-Mor, 2019). The differences seen in the list of prevalent serovars may arise due to differences in food consumption habits, geographical locations and climate which may give certain serovars advantages over others. As well, a larger sample size of *Salmonella* strains in Saudi Arabia may reveal a different distribution of serotypes across the country.

The main *Salmonella* serovars identified in this study, *S*. Enteritidis and *S*. Typhimurium, were also isolated from non-fecal samples such as the blood. This may suggest that these strains, especially *S*. Enteritidis, are more invasive and have the potential to cause systemic infection compared to the other serotypes. However, this conclusion has to be confirmed with a larger sample size that includes regions from across the country as it is possible that this observation is only being seen since the aforementioned serotypes are more frequently isolated. As well, we are unable to determine if any of the isolates caused iNTS at this time as there is no data available on the presence of these strains in Saudi Arabia or in the region.

Traditionally, the prevailing view was that SPI-1 was mainly associated with intestinal invasion of epithelial cells, while SPI-2 was involved in systemic infection (Hansen-Wester and Hensel, 2001). The observation that there is a certain level of cross talk between the two secretion systems has led to an understanding that both SPI-1 and SPI-2 are important virulence factors during the process of infection (Moest and Méresse, 2013). However, the majority of the data has been derived from cell culture models or animal models of infection. Relatively, the role of SPI-1 and SPI-2 in human infections is still not clearly defined. The present study was an attempt to determine if SPI-1 and SPI-2 are present in clinical isolates associated with human disease in Saudi Arabia. Our data clearly show that SPI-1 was present in the majority of the *Salmonella* strains and that strains missing SPI-1 genes only had a single missing gene like *sopE2*. The absence of the aforementioned SPI-1 genes indicates that these strains are capable of forming a fully functional SPI-1 T3SS with a minimal effect, if any, on virulence since the effector proteins encoded by these genes are known to have important but redundant roles (Zhang et al., 2018). On the other hand, the SPI-2 gene *sifA* was not detected in several isolates, while the *ssaR* gene (SPI-2) was present in all the samples. This demonstrates that an intact SPI-2 T3SS was likely present in the *sifA* deficient isolates, since this gene is located outside SPI-2 and plays a role in the formation of *Salmonella* induced filaments and maintenance of the *Salmonella* containing vacuole (Stein et al., 1996). However, *sifA* mutants are known to be highly attenuated in systemic infection and intracellular replication in mice (Beuzón et al., 2000; Rajashekar et al., 2014), thereby suggesting that isolates with these genotypic characteristics might have a reduced systemic infection potential in humans, though the exact role of SPI-2 in human infection remains to be elucidated. Our SPI-1 results are not in agreement with a study in China which indicated that SPI-1 was not required for human gastroenteritis since two *S*. Senftenberg strains isolated from a food-borne disease outbreak were SPI-1 deficient (Hu et al., 2008). However, our finding that SPI-2 genes, with the exception of *sifA*, were present in all the test strains imply that SPI-2 is associated with human disease and are in accordance with the findings of the former study (Hu et al., 2008). Another noteworthy observation was that 97% (28/29) of the *sifA* deficient strains were only isolated from stool samples (data not shown), implying that SPI-2 is important in human infection. However, a detailed analysis of the exact role of the individual SPI-1 and SPI-2 effectors during human enterocolitis remains to be elucidated.

Antibiotic resistance has been reported in *Salmonella* species since the 1990's (Threlfall et al., 2000) and it appears that resistance is on a rise (Frye and Jackson, 2013). Our findings indicate that antibiotic resistance among the strains tested were the highest to nitrofurantoin, followed by ampicillin, cefalotin, tigecycline, ciprofloxacin, and trimethoprim/sulfamethoxazole. The rate of resistance to ampicillin is in agreement with a previous report from the U.S. where resistance was 14% among 23,585 human *Salmonella* isolates (Frye and Jackson, 2013). However, Ciprofloxacin resistance in our study (8%) was significantly higher and in contrast to the findings of this report (0.11%), but in agreement with data from countries in the Arab League (Moghnieh et al., 2018). The U.S. study did not assess resistance to Nitrofurantoin, Cefalotin or Tigecycline making the comparison with our data difficult (Frye and Jackson, 2013). Nevertheless, resistance to other antibiotics in our study like Amikacin, Gentamicin, Amoxicillin/Clavulanic acid and Ceftriaxone were similar to those reported. Additionally, our data also suggest that *S*. Agona, *S*. Give, *S*. Havana, *S*. Kentucky, and *S*. Minnesota are MDR strains, which is in line with similar observations by others for serotypes like *S*. Agona (Kuang et al., 2014; Irfan et al., 2015), *S*. Kentucky (Le Hello et al., 2013; Seiffert et al., 2014), and *S*. Minnesota (Campos et al., 2018), while it is rare for *S*. Give and *S*. Havana (Bekal et al., 2013).

Our observation that 70% of our test strains conferring β-lactam resistance contained the *bla*_TEM−1_ gene while very few strains contained *bla*_PSE−1_/*bla*_CARB−2_ and *bla*_OXA−1_ is also supported by a report from the UK where the authors examined 3,491 *Salmonella* isolates (Neuert et al., 2018). These results are partially in agreement with a study performed in Shanghai where β-lactam resistance was distributed between the *bla*_TEM_ and *bla*_OXA_ genes (Wang et al., 2017). However, our results are in contrast to data from France where the *bla*_PSE−1_ gene was predominantly associated with β-lactam resistance whereas the *bla*_TEM_ gene was detected at low levels (Weill et al., 2006). The latter two studies may not be an accurate comparison with our work since they focused on *S*. Typhimurium. The presence of CTX-M-type extended spectrum β-lactamases was detected in only 2 isolates (*S*. Give and *S*. Kentucky) in our study and is similar to what has been previously published (Wang et al., 2017; Neuert et al., 2018). Our finding that the MDR carbapenem *S*. Kentucky strain contained the *bla*_OXA−48_ gene is a significant finding and the first report of its kind in Saudi Arabia. The association of this strain with human infections is concerning due to the fact that an increase in the number of such strains can lead to new challenges faced by physicians who encounter patients infected with this strain. Similar findings associated with *S*. Kentucky are rare, but have been reported previously in other countries (Le Hello et al., 2013; Seiffert et al., 2014), implying that this serotype may be an emerging MDR pathogen and may require increased surveillance in order to minimize outbreaks related to such strains. The association of mutations with the *gyrA* and *parC* genes when associated with ciprofloxacin resistance is evident from our data since an MIC >4 μg/ml resulted in two point mutations in the *gyrA* gene (codons 83 and 87) and *parC* gene (codons 57 and 80) as shown elsewhere (Weill et al., 2006; Wang et al., 2017; Neuert et al., 2018).

Taken together, we have established that the CTS system can be used to rapidly serotype *Salmonella* species in Riyadh. We have demonstrated that *S*. Enteritidis and *S*. Typhimurium are the most prevalent serovars associated with human infection in Riyadh (Saudi Arabia) and that *S*. Livingstone, *S*. Kentucky and *S*. Poona are also significant among a list of 36 serovars. Our data further suggest that important virulence factors like SPI-1 and SPI-2 were present in these isolates and strains lacking *sifA* were not associated with systemic infection. However, extensive work is still required to determine the roles of these T3SS in human enterocolitis and systemic infection. We also report the finding of a unique MDR resistant *S*. Kentucky isolate conferring carbapenem resistance. Further, our results indicate that there is a rising trend of ciprofloxacin and nitrofurantoin resistance among *Salmonella* species in the region.

## Data Availability

The raw data supporting the conclusions of this manuscript will be made available by the authors, without undue reservation, to any qualified researcher.

## Author Contributions

TD, AM, SA, HB designed the study. TD, AM, and SA assisted in sample collection. MA did the majority of the experimental work, while MAZ was instrumental in MLST PCR and confirming carbapenem resistance. TD analyzed all the serotyping data. MFA analyzed all the MLST Data and the sequencing results. MD and TD led the AMR work and analysis. TD and WK led the detection of SPI-1 and SPI-2 and analyzed the results. TD wrote the manuscript with major contributions from MA, MD, and WK. AM, SA, and HB critically read the manuscript with important feedback and input. All the authors contributed to, read and approved the final manuscript.

### Conflict of Interest Statement

The authors declare that the research was conducted in the absence of any commercial or financial relationships that could be construed as a potential conflict of interest.
